# Impact of immunohistochemistry-based subtyping of GATA3, CK20, CK5/6, and CK14 expression on survival after radical cystectomy for muscle-invasive bladder cancer

**DOI:** 10.1038/s41598-021-00628-5

**Published:** 2021-10-27

**Authors:** Tanan Bejrananda, Kanet Kanjanapradit, Jirakrit Saetang, Surasak Sangkhathat

**Affiliations:** 1 Urology Unit, Division of Surgery, Prince of Songkla University, Hatyai, Songkhla 90110 Thailand; 2 Division of Pathology, Prince of Songkla University, Hatyai, Songkhla 90110 Thailand; 3 EZ-Mol-Design Laboratory, Faculty of Medicine, Prince of Songkhla University, Hat Yai, Songkhla 90110 Thailand; 4 Division of Surgery, Prince of Songkla University, Hatyai, Songkhla 90110 Thailand; 5 Translational Medicine Research Center, Prince of Songkla University, Hatyai, Songkhla 90110 Thailand

**Keywords:** Cancer, Biomarkers, Medical research

## Abstract

Molecular subtyping of muscle-invasive bladder cancer (MIBC) predicts disease progression and treatment response. However, standard subtyping based on transcriptomic analysis is relatively expensive. This study tried to use immunohistochemistry (IHC) to subtype MIBC based on GATA3, CK20, CK5/6, and CK14 protein expression. The IHC-based subtypes in MIBC subtypes were classified as luminal (GATA3^+^ CK5/6^−^, 38.6%), basal (GATA3^−^CK5/6^+^, 12.9%), mixed (GATA3^+^ CK5/6^+^, 37.9%), and double-negative (GATA3^−^CK5/6^−^, 10.6%) in 132 MIBC patients. All individual markers and clinicopathological parameters were analyzed against treatment outcomes after radical cystectomy. The mean patient age was 65.6 years, and the male to female ratio was 6.8:1. Positive IHC expression of GATA3, CK20, CK5/6, and CK14 were 80.3%, 50.8%, 42.4%, and 28.0%, respectively. Only GATA3 and CK5/6 were significantly associated with survival outcome (*p* values = 0.004 and 0.02). The mixed subtype was significantly better in 5-year OS at 42.8%, whereas the double-negative subtype had the worst prognosis (5-year OS 7.14%). The double-negative subtype had a hazard ratio of 3.29 (95% CI 1.71–6.32). Subtyping using GATA3 and CK5/6 was applicable in MIBCs, and patients with the double-negative subtype were at the highest risk and may require more intensive therapy.

## Introduction

Urinary bladder cancer is among the top 10 most frequent cancers and one of the most common causes of cancer-related deaths in humans with an estimated 500,000 new cases and 200,000 deaths per year worldwide^1,2^. In Thailand, the estimated incidence of bladder cancer from 2013 to 2015 was approximately 4.0 cases/100,000 population-years^3^. Bladder cancer is a disease with high heterogeneity in its pathology and clinical presentation. Urothelial carcinoma accounts for more than 90% of bladder cancers^4^. Generally, urothelial carcinoma is categorized into non-muscle-invasive bladder cancer (NMBC) and muscle-invasive bladder cancer (MIBC) according to bladder wall invasion^5^. While NMBC generally has a low risk of distant metastasis and better outcomes, MIBC is more aggressive and is more likely to metastasize. MIBC usually requires intensive management, which includes radical cystectomy with perioperative chemotherapy^6,7^. Despite complete surgery and adjuvant therapy, the 5-year overall survival (OS) of MIBC is approximately 36%^8^.

MIBC has a high level of genomic instability, and *TP53* mutations are the most common genetic abnormalities found in the tumor tissue^9^. Studies of HER2/neu, E-cadherin, p53, and p16 expression in MIBC tumor tissue using immunohistochemistry (IHC) have been reported^10^. However, none of these markers has been applied in clinical practice. Recently, comprehensive mRNA expression profiles in bladder cancer were used to categorize MIBC into various molecular subtypes^4,11–13^. The primary molecular subtypes of MIBC are the luminal and basal subtypes^13^, which are similar to the initially reported molecular breast cancer subtypes^14,15^. Although further studies have extended the molecular classification of MIBC into 5 or 6 subtypes^16,17^, the luminal and basal subtypes remain the fundamental types.

Intrinsic subtypes of bladder cancer have demonstrated increased utility in predicting treatment outcomes, especially in patients with MIBC who undergo radical cystectomy followed by adjuvant chemotherapy^18^. The established molecular subtypes proposed in previous studies were primarily established by high-throughput molecular technology, especially transcriptomic analysis. Traditional IHC techniques are not technology-dependent, and hence it is more feasible to classify subtypes at the protein level using IHC. Discovery of potential IHC markers that can stratify molecular subtypes of MIBC may be useful in the prediction of disease progression. Previous studies have reported a correlation between mRNA expression profiles and IHC staining results in luminal (CK20 expression) and basal (CK5/6 expression) subtypes^11^ and also confirmed that GATA3 and CK5/6 expression by IHC may also identify these two subtypes with greater than 90% accuracy according to a meta-analysis^19^. Another study revealed that the basal/squamous-like subtype was correlated with poor clinical outcome^20^, as was decreased GATA3 expression^21^. In this study, GATA3, CK20, CK5/6, and CK14 staining was selected to be tested against clinical outcomes with respect to survival after a radical cystectomy. This study aimed to determine IHC markers or patterns that may predict prognosis in patients with MIBC.

## Results

### Demographic and clinicopathological data

**Figure 1:**
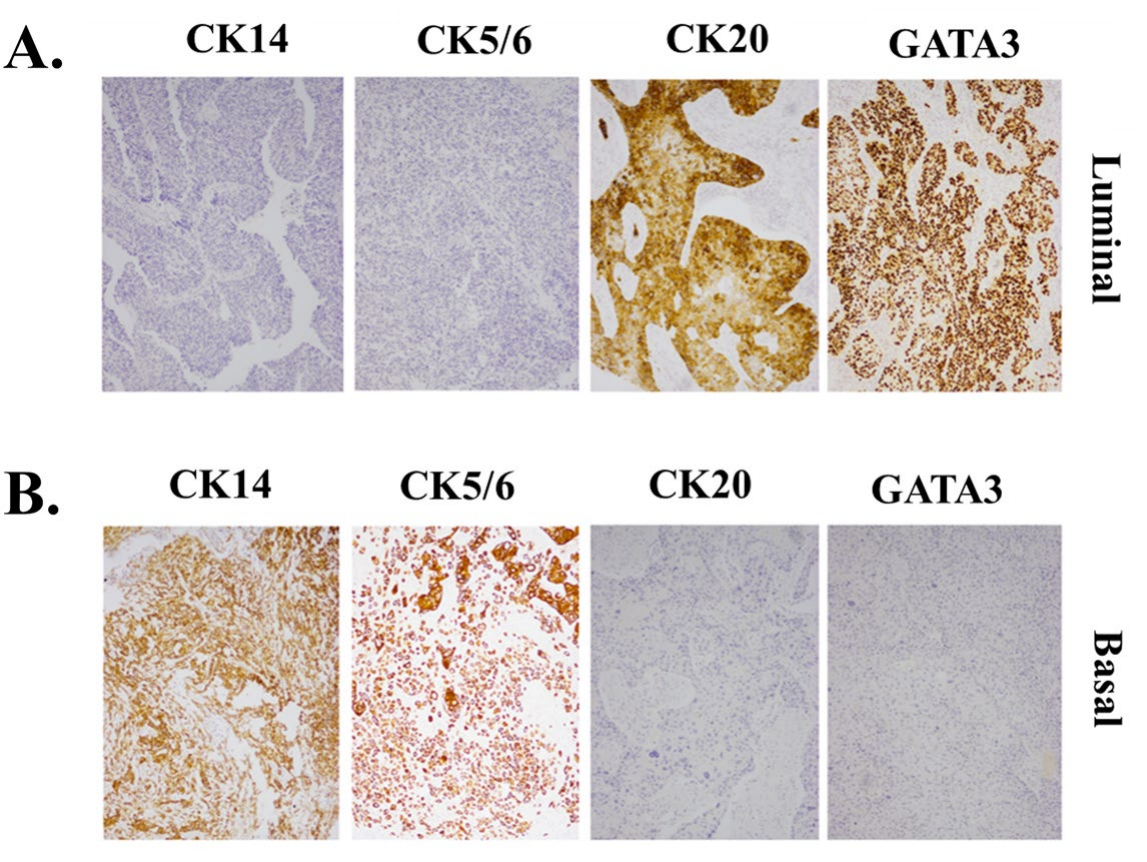
Immunohistochemical staining of MIBC tissues for GATA3, CK20, CK5/6, and CK14. (**A**) Luminal type, (**B**) Basal type.

This study included 132 patients with MIBC who underwent radical cystectomy during the study period. Their mean age was 65.6 years, and the male to female ratio was 6.8:1. The demographic characteristics of the patients and immunoreactivity for each IHC marker are summarized in Table 1. Two patients who died at 3 and 7 days after surgery were considered to have operative mortality and were excluded from the survival analysis. As of January 2021, the median follow-up duration was 125 months (interquartile range 103–154 months). The median OS time was 12.2 months (interquartile range 4.7, 46.4 months), and the 5-year OS was 27.0% (95% CI 19.6%–35.0%). IHC showed positivity for GATA3, CK5/6, CK20, and CK14 with kappa value between 0.799–0.908 (93.2–96.2% agreement) (Table 2). The immunostains for GATA3, CK5/6, CK20, and CK14 showed positive results with 80.3%, 50.8%, 42.4%, and 28.0% of cases, respectively (Fig. 1). GATA3 and CK5/6 immunopositivity was significantly associated with OS by log-rank analysis (Table 1). Twenty-six cases received a median of 3 cycles of adjuvant chemotherapy. GATA3 expression was significantly inversely correlated with pT stage progression.

**Table 1:** Clinicopathological features of the 132 patients who underwent radical cystectomy.

Variable	Value (n, %)	5-year OS (95% CI)	Log-rank *p* value
**Age (years)**			
Mean (SD)	65.6 (9.3)	–	
**Sex**			0.56
Male	115 (87.1%)	25.7 (17.9–34.2)	
Female	17 (12.9%)	35.3 (14.5–57.0)	
**ECOG status**			0.18
0	29 (22.0%)	39.2 (21.6–56.5)	
1	103 (78.0%)	23.7 (15.9–32.6)	
**T stage**			< 0.01
T1	21 (15.9%)	68.6 (50.0–94.1)	
T2	23 (14.7%)	44.0 (30.2–73.1)	
T3	41 (31.1%)	17.1 (8.7–33.5)	
T4	47 (35.6%)	6.4 (2.1–19.1)	
**N stage**			
N0	90 (68.2%)	33.7 (23.8–43.8)	< 0.01
N1	24 (18.2%)	12.2 (4.5–24.1)	
N2	15 (11.4%)		
N3	3 (2.3%)		
**M stage**			NA
M0	128 (97.7%)	26.9 (19.4–34.9)	
M1	3 (2.3%)	NA	
**Tumor grade**			0.04
Low	7 (5.30%)	85.7 (33.4–97.9)	
High	125 (94.7%)	23.7 (16.5–31.7)	
**Chemotherapy**			0.80
No	106 (80.3%)	27.9 (19.5–37.0)	
Yes	26 (19.7%)	23.1 (9.4–40.3)	
**Diversion**			0.03
Ileal conduit	123 (93.2%)	24.0 (16.6–32.1)	
Neobladder	9 (6.8%)	66.7 (28.2–87.8)	
**LVI**			< 0.01
Negative	52 (39.4%)	40.0 (26.3–53.3)	
Positive	80 (60.6%)	18.8 (11.1–28.2)	
**CK20**			0.45
Negative	76 (57.6%)	25.3 (16.2–35.5)	
Positive	56 (42.4%)	29.7 (18.0–42.4)	
**CK5/6**			0.02
Negative	65 (49.2%)	16.2 (8.3–26.5)	
Positive	67 (50.8%)	37.8 (26.2–49.3)	
**CK14**			0.63
Negative	95 (72.0%)	26.0 (17.5–35.4)	
Positive	37 (28.0%)	30.6 (16.6–45.7)	
**GATA3**			< 0.01
Negative	26 (19.7%)	16.1 (5.9–30.9)	
Positive	106 (80.3%)	30.5 (21.6–39.9)

**Table 2:** Immunopositivity of the four markers analyzed and their correlation with clinicopathological parameters.

	All	GATA3	CK5/6	CK20	CK14
**Positive staining (%)**	132	101 (76.5%)	67 (50.8%)	56 (42.4%)	37 (28.0%)
**Mean age of positive cases (SD)**		64.7 (9.3)	64.7 (9.1)	64.8 (8.7)	65.7 (9.3)
**Gender**					
Male (%)	115 (87.1%)	91 (90.1%)	56 (83.6%)	50 (89.3%)	30 (81.1%)
Female (%)	17 (12.9%)	10 (9.9%)	11 (16.4%)	6 (10.7%)	7 (18.9%)
**ECOG status, n (%)**					
0	29 (22.0%)	25 (24.7%)	13 (19.4%)	13 (23.1%)	8 (21.6%)
1	103 (78.0%)	76 (75.3%)	54 (80.6%)	43 (76.8%)	29 (78.3%)
**T stage, n (%)**					
pT1	21 (15.9%)	20 (19.8%)*	12 (17.9%)	14 (25.0%)*	0 (0.0%)*
pT2	23 (17.4%)	20 (19.8%)	14 (20.9%)	13 (23.2%)	8 (21.6%)
pT3	41 (31.1%)	28 (27.7%)	20 (29.9%)	10 (17.9%)	18 (48.7%)
pT4	47 (35.6%)	33 (32.6%)	21 (31.3%)	19 (33.9%)	11 (29.7%)
**N stage, n (%)**					
N0	90 (68.2%)	69 (68.3%)	47 (70.2%)	41 (73.2%)	27 (73.0%)
N1	24 (18.2%)	19 (18.8%)	12 (17.9%)	7 (12.5%)	7 (18.9%)
N2	15 (11.4%)	11 (10.9%)	7 (10.5%)	6 (10.7%)	2 (5.4%)
N3	3 (2.3%)	2 (2.0%)	1 (1.5%)	2 (3.6%)	1 (2.3%)
**M stage, n (%)**					
M0	128 (97.7%)	98 (98.0%)	65 (98.5%)	54 (98.2%)	35 (97.2%)
M1	3 (2.3%)	2 (2.0%)	1 (1.5%)	1 (1.8%)	1 (2.8%)
**Tumor grade, n (%)**					
Low	7 (5.30%)	6 (5.9%)	6 (9.0%)	3 (5.4%)	0 (0.0%)
High	125 (94.7%)	95 (94.1%)	61 (91.0%)	53 (94.6%)	37 (100.0%)
**LVI, n (%)**					
Negative	52 (39.4%)	41 (40.6%)	26 (38.8%)	25 (44.6%)	11 (29.7%)
Positive	80 (60.6%)	60 (59.4%)	41 (61.1%)	31 (55.4%)	26 (70.3%)

*p value < 0.05 when distribution between positive cases and among all cases was compared; LVI: lymphovascular invasion.

**Figure 2:**
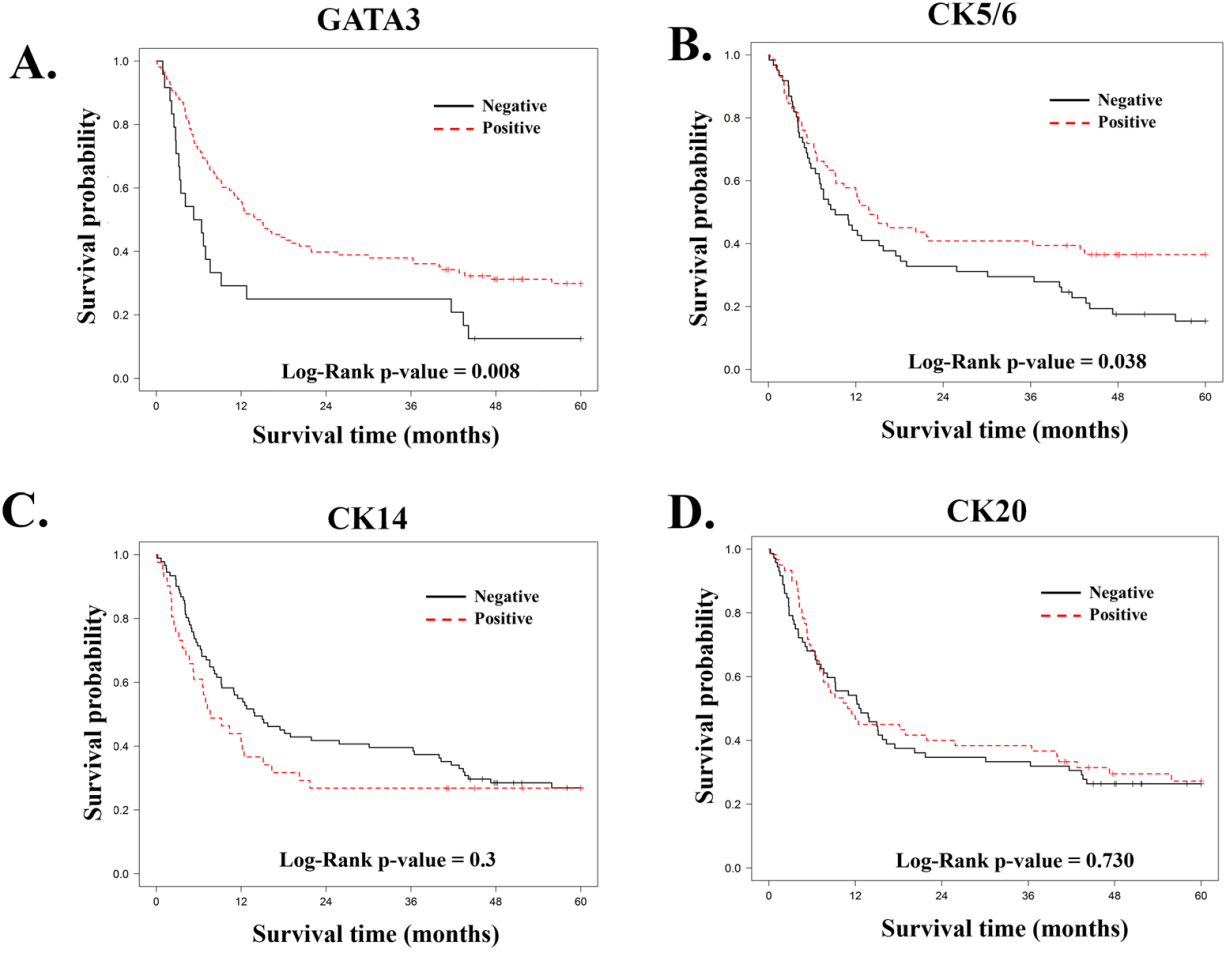
(**A**–**D**) Kaplan–Meier curves demonstrate the survival probability in 132 patients with MIBC according to marker expression by IHC; GATA3 (**A**), CK5/6 (**B**), CK14 (**C**), and CK20 (**D**).

According to the Kaplan–Meier survival analysis, significant differences in outcomes with respect to OS were demonstrated among cases with positive GATA3 staining (*p* = 0.008) and in cases with positive CK5/6 staining (*p* = 0.038). The other markers did not show significant prognostication value for survival. The Kaplan–Meier survival curves for GATA3, CK5/6, CK14, and CK20 are depicted in Fig. 2.

**Figure 3:**
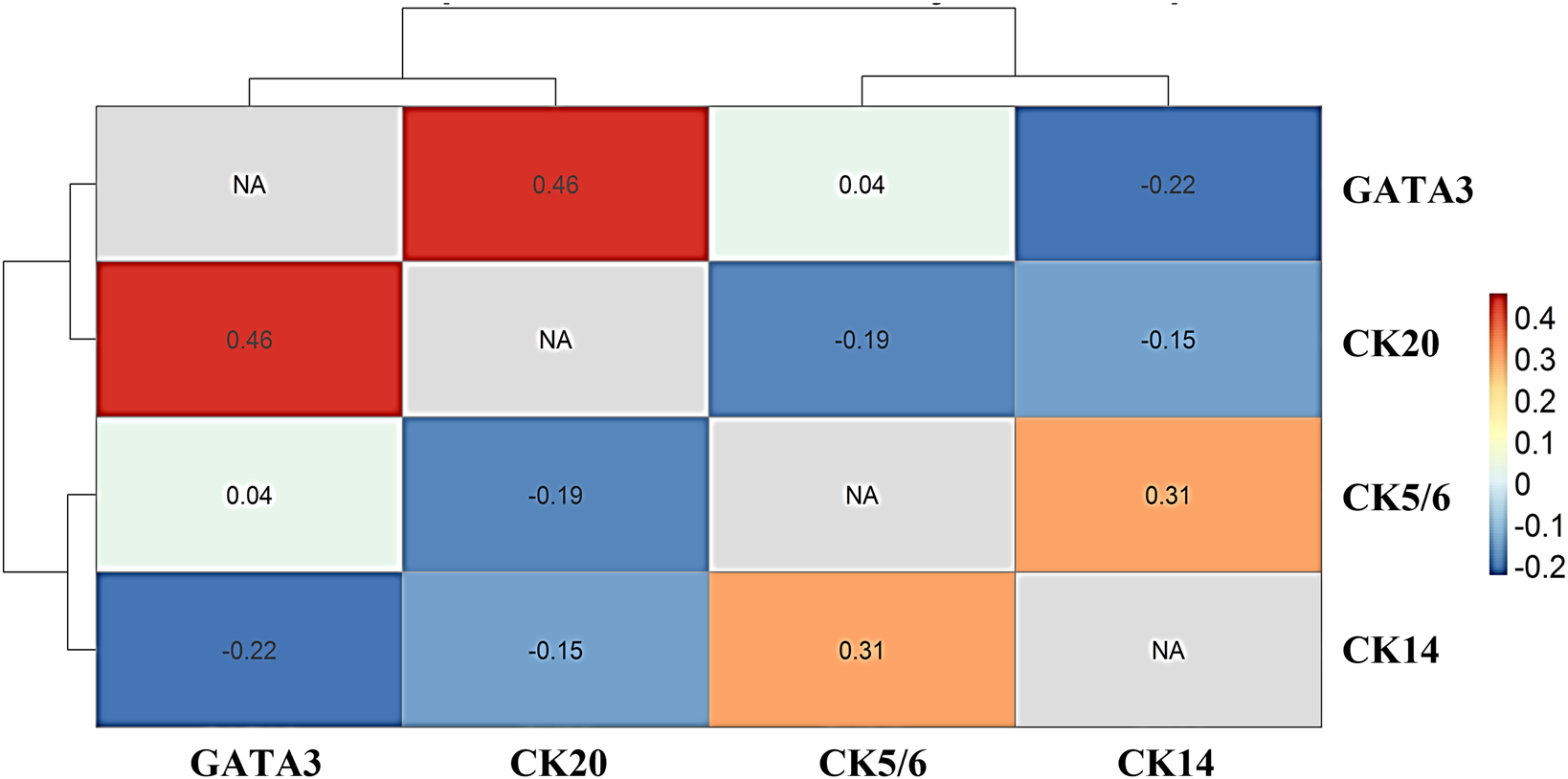
Correlation heatmap of GATA3, CK20 (basal-like markers), CK5/6, and CK14 (luminal-like markers) expression by IHC.

The correlation between each individual marker was evaluated by Pearson correlation test. As showed in Table 2, the significant association of GATA3, CK5/6, and CK20 was found only in pathological stage 1 of patients. When the correlation between markers in the basal and luminal subtypes was assessed, moderate correlation was observed between GATA3 and CK20 expression, which indicated that the basal-like subtype was demonstrated by Pearson correlation at 0.46 (*p* = 0.022). The analysis showed small correlation between the luminal-like subtype markers, CK5/6 and CK14; 0.31 (*p* = 0.048) (Fig. 3).

As GATA3 and CK5/6 were the only two markers representing different subtypes that were significantly associated with survival, we elected to categorize our cases into four groups according to those two markers: luminal-like (GATA3^+^ and CK5/6^−^), basal-like (GATA3^−^ and CK5/6^+^), mixed (GATA3^+^ and CK5/6^+^), and double-negative (GATA3^−^ and CK5/6^−^) subtypes. By this definition, the luminal-like, basal-like, mixed, and double-negative subtypes were observed in 38.6%, 12.9%, 37.9%, and 10.6% of cases, respectively. Associations between each subtype and clinicopathological factors including survival outcomes are displayed in Table 3. The double-negative subtype was significantly associated with higher incidence of pT4 disease.

**Table 3:** Patient characteristics and classification by IHC subtype according to GATA3 and CK5/6 expression.

	Double-neg	Luminal-like	Basal-like	Mixed	*p* value**
**Total (%)**	14 (10.6)	51 (38.6)	17 (12.9)	50 (37.9)	–
**Mean age (SD)**	70.2 (6.0)	65.3 (10.1)	66.6 (10.7)	64.1 (8.6)	0.18
**Gender, n (%)**					
Male	12 (85.7)	47 (92.2)	12 (70.6)	44 (88.0)	0.15
Female	2 (14.3)	4 (7.8)	5 (29.4)	6 (12.0)	
**ECOG status, n (%)**					0.29
0	1 (7.1)	15 (29.4)	3 (17.7)	10 (20.0)	
1	13 (92.9)	36 (70.6)	14 (82.3)	40 (80.0)	
**Urinary diversion type**					0.98
Ileal conduit	13 (92.9)	47 (92.2)	16 (94.1)	47 (94.0)	
Neobladder	1 (7.1)	4 (7.8)	1 (5.9)	3 (6.0)	
**T stage, n (%)**					0.24
T1	0 (0.0)	9 (17.6)	1 (5.9)	11 (22.0)	
T2	0 (0.0)	9 (17.6)	3 (17.6)	11 (22.0)	
T3	6 (42.9)	15 (29.4)	7 (41.2)	13 (26.0)	
T4	8 (57.1)	18 (35.3)	6 (35.3)	15 (30.0)	
**N stage, n (%)**					0.71
N0	11 (78.6)	32 (62.8)	10 (58.8)	37 (74.0)	
N1	1 (7.1)	11 (21.6)	4 (23.5)	8 (16.0)	
N2	1 (7.1)	7 (13.7)	3 (17.7)	4 (8.0)	
N3	1 (7.1)	1 (2.0)	0 (0.0)	1 (2.0)	
**M stage, n (%)**					0.55
M0	13 (92.9)	50 (98.0)	17 (100.0)	48 (98.0)	
M1	1 (7.1)	1 (2.0)	0 (0.0)	1 (2.0)	
**Tumor grade, n (%)**					0.25
Low	0 (0.0)	1 (2.0)	1 (5.9)	5 (10.0)	
High	14 (100.0)	50 (98.0)	16 (94.1)	45 (90.0)	
**Margin, n (%)**					0.57
Negative	11 (78.6)	46 (90.2)	16 (94.1)	43 (86.0)	
Positive	3 (21.4)	5 (9.8)	1 (5.9)	7 (14.0)	
**LVI, n (%)**					0.97
Negative	5 (35.7)	21 (41.2)	6 (35.3)	20 (40.0)	
Positive	9 (64.3)	30 (58.8)	11 (64.7)	30 (60.0)	
**CK20, n (%)**					< 0.01
Negative	12 (85.7)	17 (33.3)	16 (94.1)	31 (62.0)	
Positive	2 (14.3)	34 (66.7)	1 (5.9)	19 (38.0)	
**CK14, n (%)**					< 0.01
Negative	12 (85.7)	48 (94.1)	4 (23.5)	31 (62.0)	
Positive	2 (14.3)	3 (5.9)	13 (76.5)	19 (38.0)	
**5-Year OS (%) (95% confidence interval)**	7.14 (0.4–27.5)	18.9 (9.2–31.1)	23.5 (7.3–24.9)	42.8 (28.9–56.1)	< 0.01

*p value by Chi-square or Fisher’s exact test; ECOG status: Eastern Cooperative Oncology Group performance status; LVI: lymphovascular invasion; OS: overall survival.

CK20 immunopositivity, a marker of the luminal molecular subtype, was significantly associated with the GATA3-defined luminal subtype (*p* < 0.01), whereas CK14 positivity was significantly associated with the CK5/6-defined basal subtype (*p* < 0.01). In the 50 mixed subtype (GATA3^+^ and CK2/5^+^) cases in this study, an equal number of cases with CK20 and CK5/6 positivity was found (Table 3).

When clinicopathological parameters and IHC subtypes were analyzed against survival in a univariable Cox hazard model, tumor stage (pT and N), lymphovascular invasion, pathologic grade, loss of GATA3 immunoreactivity, and loss of CK5/6 immunoreactivity were significantly associated with poorer survival outcomes. Considering subtyping, while patients with the mixed subtype had the lowest risk, which was followed by patients with the luminal-like and basal-like subtypes, those with the double-negative subtype had the highest crude HR. In the multivariable analysis by stepwise Cox hazard regression, N stage (N > 0) and the double-negative subtype were significantly associated with higher risk (model *p* = 0.0001) (Table 4).

**Table 4:** Univariable and multivariable regression analyses of clinical outcomes in 132 patients with MIBC.

Factor	Univariable analysis		Multivariable analysis	
	Crude HR (95% CI)	*p* value	Adj. HR (95% CI)	*p* value
**T stage**				
pT1	1.00 (reference)	< 0.01		
pT2	1.61 (0.65–3.95)			
pT3	5.15 (2.36–11.19)			
pT4	6.50 (3.01–14.02)			
**N stage**				
N0	1.00 (reference)	< 0.01	1.00 (reference)	0.02
N1	1.78 (1.07–2.96)		1.84 (1.09–3.13)	< 0.01
N2	2.58 (1.43–4.66)		2.63 (1.44–4.78)	
N3	5.34 (1.63–16.58)		4.45 (1.35–14.68)	0.01
**LVI (positive)**	1.94 (1.27–2.96)	< 0.01		
**Grade (high grade)**	3.16 (1.00–10.00)	0.02		
**GATA3 (negative)**	1.87 (1.20–2.90)	< 0.01		
**CK5/6 (negative)**	1.57 (1.06–1.35)	0.03		
**CK20 (negative)**	1.16 (0.78–1.75)	0.45		
**CK14 (negative)**	0.89 (0.58–1.39)	0.63		
**Mixed subtype**	0.52 (0.34–0.81)	< 0.01		
**Basal subtype**	1.39 (0.79–2.46)	0.25		
**Luminal subtype**	1.18 (0.79–1.76)	0.43		
**Double-negative**	2.24 (1.27–3.96)	< 0.01		
**Subtypes**		< 0.01		
Mixed	1 (reference)		(Reference)	1
Luminal	1.66 (1.03–2.68)		1.66 (0.86–3.21)	0.13
Basal	2.01 (1.06–3.81)		1.60 (0.99–2.60)	0.05
Double-negative	3.12 (1.63–5.92)		3.29 (1.71–6.31)	< 0.01

Crude HR: crude hazard ratio; adj. HR: adjusted hazard ratio; 95% CI: 95% confidence interval; LVI: lymphovascular invasion, Univariate and multivariable Cox regression analysis of overall survival (Cox proportional hazards regression model).

### Association between molecular subtypes and survival outcomes

**Figure 4:**
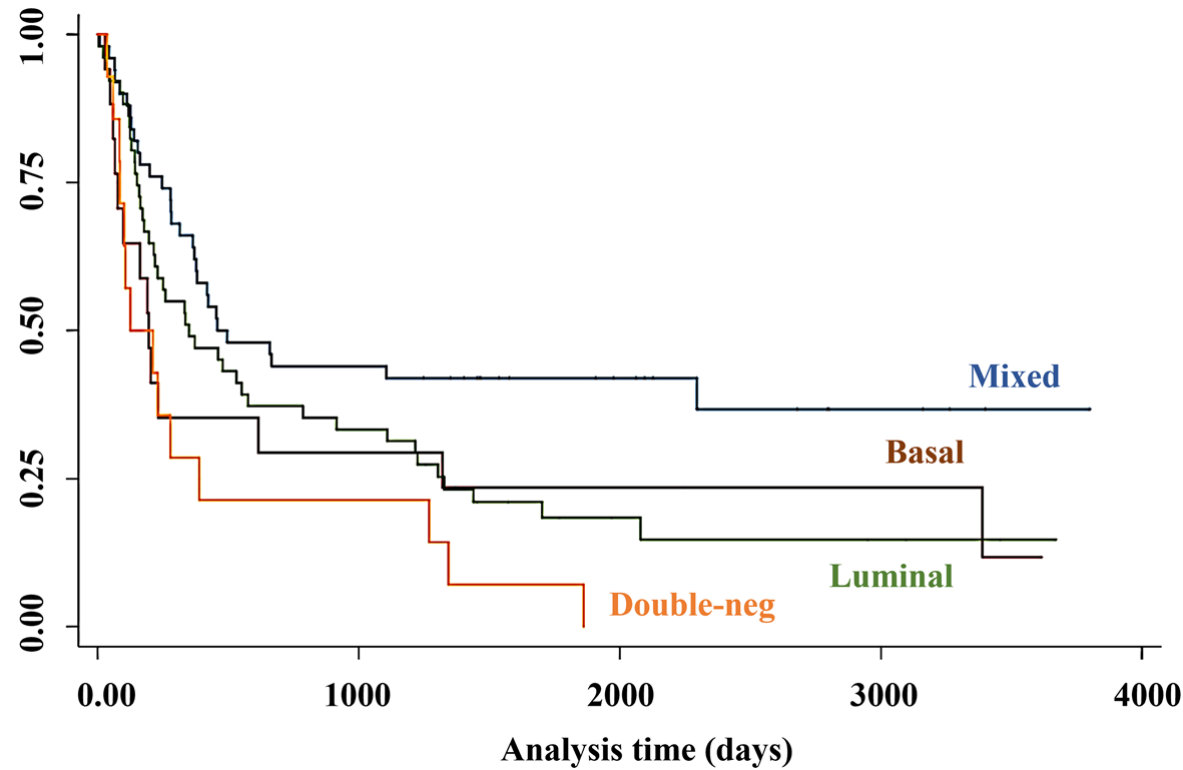
Kaplan–Meier survival curves of patients with different molecular subtypes of muscle-invasive bladder cancer according to their immunoexpression of four markers.

Kaplan–Meier curves compare the survival probability of 132 patients with MIBC following radical surgery (Fig. 4). The 5-year OS rates of patients with the mixed, basal, luminal, and double-negative subtypes were 42.5% (95% CI 28.9–56.1%), 23.5% (7.3–44.9%), 18.9% (9.2–31.1%), and 7.1% (0.4–27.5%), respectively.

In the 50 MIBCs of mixed subtype (GATA3^+^ and CK5/6^+^), if CK20 and CK14 were added to the subcategorization criteria, which means that mixed subtype cases with positive CK20 were reclassified as luminal, whereas mixed subtype cases with positive CK14 were reclassified as basal. The univariable hazard model did not improve. Using the mixed subtype as a reference, the HRs (and 95% CIs) for the luminal, basal, and double-negative subtypes were 1.17 (95% CI 0.62–2.20), 1.54 (0.76–3.10), and 2.64 (1.22–5.73) respectively.

## Discussion

The molecular subtyping of bladder cancer represents disease heterogeneity. Previous gene expression profiling has revealed that MIBCs can be subcategorized into at least three intrinsic subtypes including the luminal, basal, and double-negative subtypes^22^. The characteristics of luminal tumors include high expression of markers (GATA3, CK20, and uroplakin 2) of terminally differentiated urothelial cells, which are also known as umbrella cells^13^. As umbrella cells have shorter longevity than basal cells, they are less susceptible to genomic alterations but usually exhibit greater changes in their chromatin landscape. Basal-like bladder cancer cells express biomarkers of mesenchymal stem cells (CK5/6 and CK14) and exhibit some squamous and sarcomatous features in tumor tissue^23^. Recent studies have suggested that GATA3 and CK5/6 expression can identify molecular subtypes in 80–90% of cases^19,22^. In our study, 62% of cases could be clearly categorized into luminal, basal, or double-negative subtypes based on GATA3 and CK5/6 expression, and only these two markers were associated with survival outcome. Loss of expression of either GATA3 or CK5/6 was associated with poorer survival probability, whereas loss of expression of both markers was a strong predictor of poor outcome. Although a significant association was observed between CK20 and GATA3 expression and between CK14 and CK5/6 expression, the addition of CK20 and CK14 to the criteria to categorize the subtypes did not appear to improve survival prediction.

MIBCs expressing GATA3 exhibited less aggressive characteristics and were associated with significantly better survival. GATA3, also known as GATA3 binding protein, is a transcription factor that regulates the expression of genes that function in the luminal differentiation of breast and urothelial epithelium^24,25^. In addition, GATA3 is expressed in T-lymphocytes, the central nervous system, and erythrocytes^26^. In breast cancers, reduced GATA3 expression was reported in the triple-negative subtype^27^. In urothelial cell line models, the loss of GATA3 expression promoted tumor cell migration and invasion via upregulation of oncogenes^28,29^. Several clinical studies of GATA3 in bladder cancer have been conducted^30–32^. Loss of GATA3 expression was associated with high-grade cancer^32^, and patients with GATA3-negative bladder cancer had poorer survival outcomes in most studies^30,31,33^. Taken together, those reports and our data demonstrate that GATA3 is a promising biomarker of MIBC. Another luminal marker evaluated in this study, CK20, has been reported to be correlated with higher tumor grade and stage in papillary urothelial carcinoma^34^. However, our study did not reveal a significant association between CK20 and any clinicopathological factor or survival outcome.

CK5/6 is a cytokeratin expressed in a squamous epithelial lineage and is generally used as a marker of squamous differentiation, which indicates the basal subtype^35^. Expression of CK5/6 in urothelial carcinoma was associated with poorer survival in several reports^35,36^. In contrast, some reports also demonstrated that loss of CK5/6 expression was associated with decreased survival probability in patients with transitional cell carcinoma of the upper urinary tract^36,37^. In our study, loss of CK5/6 expression was associated with significantly worse survival outcome. CK14 is another marker of the basal subtype, and its expression has been reported to be negatively correlated with survival in MIBC.

Although molecular subtyping has been accepted for its correlation with disease progression and treatment outcome in MIBC, RNA expression profiling is not a widely available technology. The evaluation of IHC panels for intrinsic subtype categorization of MIBC has been reported in several studies^31,36,38^. However, except for GATA3, the prognostic value of other IHC markers has remained intriguing. Variation in staining techniques and interpretation may partly explain the disparity in results. Since our study revealed significant associations between survival and GATA3 and CK5/6 expression, these markers were combined in a simple subgroup as luminal when the tumor had exclusive expression of GATA3 and as basal for exclusive CK5/6 expression. Our study also found that the double-negative subtype, which is indicated by negative staining of both markers, predicted the poorest outcome. Other combinations that have been reported in previous studies, such as CK20 with CK5/6 or CK20 with CK14, have been investigated with no interesting findings.

The limitations of our study included the small sample size and the lack of gene expression profiling to validate concordance between molecular subtypes and IHC marker expression. However, the transcriptomic profiling study using the markers from this study as a part of clustering is running and will be launched in the next year. In addition, only 20% of our patients received chemotherapy after radical cystectomy because the physical status of most patients did not allow for chemotherapy. As neoadjuvant chemotherapy is becoming a new trend in MIBC treatment, the results of our study may help in the selection of patients at a high risk for treatment failure who should receive upfront chemotherapy before definitive surgery.

## Methods

### Patients and specimens

This study included 132 patients with urinary bladder cancer who underwent radical cystectomy and who received standard adjuvant chemotherapy at Songklanagarind Hospital, Thailand from 2008 to 2016. Inclusion criteria were patients with bladder cancer aged older than 15 years who underwent surgery primarily at our institute and who completed adjuvant treatment according to the standard of the Thai Urological Association^8^. All eligible cases were reviewed for clinical stage, and their histopathology was confirmed by a pathologist who specializes in genitourinary tract pathology (KK). Staging was performed according to the TNM classification, whereas stage grouping was performed according to the eighth version of the American Joint Committee on Cancer Staging Manual. Cases without muscularis propria invasion and those with subtypes other than non-urothelial carcinoma were excluded. Clinical data were extracted from the electronic medical records of the hospital (HIS system). Data on survival status combined with the clinical follow-up records and death registry data from the Thai citizen registration system were analyzed and archived by the Cancer Unit, Songklanagarind Hospital. Cases with operative mortality were excluded from the survival analysis. The study protocol was approved by the Human Research Ethic Committee of the Faculty of Medicine, Prince of Songkla University (REC61-222-10-1). All methods were carried out in accordance with the World Medical Association Declaration of Helsinki. Informed consent was obtained from all patients or legally authorized representatives included in the study.

### IHC study by tissue microarray

Sampling of the tumor part for this pilot study was performed by a collaborative work between the attending surgeon who know the orientation of the specimen and the pathologist who examined the histopathology. Bladder carcinoma in situ and flat lesions were excluded in this study. Several areas of tumor in the same patients for the pathological morphology and selected the representative areas that have both richness in tumor cells and the morphology was like other areas in the same cases were selected for examination. Archived pathological specimens from all included cases were retrieved as formalin-fixed paraffin-embedded tissue blocks, which were then selected and prepared as 5-μm sections for a tissue microarray (TMA) using a tissue arrayer (Beecher Instruments, Silver Spring, MD, USA). Immunostaining procedures were conducted with 3 (triplicate) TMA cores per section by a pathology technician who specializes in this technique. In cases of multiple foci, all foci were selected for examinations. Subtype-specific primary antibodies used here are as follows: GATA3 (UMAB218, 1:100 dilution; OriGene, MD, USA), CK5/6 (D5/16, 1:50 dilution; Dako, Glostrup, Denmark), CK14 (OIT4A7, 1:100 dilution; OriGene), and CK20 (OTI4A, 21:50 dilution; OriGene). These antibodies were used to identify potential markers to establish molecular subtypes in the tissue sections contained in the TMA. A pathologist (KK) blinded to the clinical outcomes examined the results using a light microscope and scored all TMA sections. For mixed and/or borderline cases, the positive immunostains were interpreted especially by the consensus of two pathologists. The positivity and intensity of tumor cell nuclei stained for GATA3 and membranous or cytoplasmic staining for CK20, CK5/6, and CK14 were recorded. Staining intensity was assessed as 0 (negative; 0–10%) or 1 (positive; 10–100%).

### Statistical analysis

Categorical and continuous parameters were compared using the Chi-square test and were analyzed using the Spearman rank correlation test. The median differences between groups for non-normally distributed variables were evaluated by independent sample Kruskal–Wallis test. Differences in the percentages of IHC staining between or among comparable groups were analyzed using the Student’s *t* test and one-way analysis of variance. The hazard ratios (HRs) and 95% confidence intervals (CIs) were also calculated. In all patients who underwent radical cystectomy with adjuvant chemotherapy, the OS after radical cystectomy was calculated using the Kaplan–Meier method. Survival probabilities were estimated using the Kaplan–Meier method, whereas the log-rank test was adopted to compare survival probabilities between each variable. All variables with *p* ≤ 0.1 in the univariable analyses were entered into the multivariable regression analysis. Multivariable analyses were also performed using Cox regression. Two-sided *p* values < 0.05 were considered statistically significant. The R program (version 4.0.1) was used for statistical analyses.
